# Measuring geographic proximity and continuity with family medicine at end-of-life: Protocol for a population-level retrospective cohort study using Canadian Health Administrative Data

**DOI:** 10.1371/journal.pone.0336790

**Published:** 2025-12-04

**Authors:** Shuaib Hafid, Lawrence Mbuagbaw, Bruce Newbold, Andrew Costa, Ana Gayowsky, Peter Gozdyra, Aaron Jones, Sarina Isenberg, Erin Gallagher, Michelle Howard

**Affiliations:** 1 Department of Family Medicine, McMaster University, Hamilton, Ontario, Canada; 2 Department of Health Research Methods, Evidence, and Impact, McMaster University, Hamilton, Ontario, Canada; 3 School of Earth, Environment, & Society, McMaster University, Hamilton, Ontario, Canada; 4 ICES McMaster, Hamilton, Ontario, Canada; 5 Centre for Integrated Care, St. Joseph’s Health System, Hamilton, Ontario, Canada; 6 ICES, Toronto, Ontario, Canada; 7 Department of Medicine, University of Ottawa, Ottawa, Ontario, Canada; 8 Bruyère Health Research Institute, Ottawa, Ontario, Canada; Public Library of Science, UNITED STATES OF AMERICA

## Abstract

**Background:**

Family physicians play an important role in coordinating care for medically complex patients, especially during the end-of-life (EOL) period. While continuity of care (COC) is a routinely measured care quality indicator, the influence of geographic proximity to family physicians on EOL COC has not been studied in the Canadian context. Existing research has focused on rurality indicators instead of individual-level proximity measures.

**Objectives:**

This study objectives are to: (1) measure the association between patients’ geographic proximity to their family physician and COC during the patients’ last year of life; and (2) measure the association between geographic proximity and the number of days spent in the community and palliative homecare services referral in the last year of life, and place of death.

**Methods:**

We will conduct a population-level retrospective cohort study using linked health administrative data from ICES in Ontario, Canada, of adults who died between January 1, 2021, and December 31, 2024. Geographic proximity to the rostered family physician will be calculated in the shortest travel distance and time from the patient’s residence to the physicians primary practice location, considering road, transit, and walking infrastructure. COC will be measured using three indices: Usual Provider of Care, Modified Bice-Boxerman, and Relative Variance indices, based on outpatient visits in the last year of life. EOL outcomes will include days spent in the community, referral to palliative home care, and place of death. Multivariate regression will measure associations between proximity and outcomes, adjusting for relevant patient-level characteristics.

**Expected outcomes:**

We hypothesize that patients living closer to their family physician will experience higher COC and improved healthcare outcomes at the end of life. Findings have the potential to inform health policy and planning aimed at improving equitable geographic access to family medicine during the late stages of life.

## 1. Introduction

### 1.1. Background

Family medicine plays a critical role in healthcare provision for individuals in Ontario, especially facilitating specialist referrals and coordinating care for medically complex patients [1–4]. This relationship is relevant at the end of life as family physicians account for approximately 65% of outpatient visits during the last year of life among patients who died in Ontario, Canada, between 2013 and 2017 [5]. The same study also identified that patients receive outpatient care from multiple physicians across four unique specialties during the last year of life. The concentration of outpatient care among family medicine reinforces the belief that family physicians are responsible for most outpatient end-of-life (EOL) care, by potentially coordinating their patients’ care across different providers.

Continuity of care (COC) is also foundational to high-quality healthcare provision, specifically for patients with medical complexity such as those at the end of life. Haggerty et al. defined continuity of care as “the degree to which a series of discrete healthcare events is experienced as coherent and connected and consistent with the patient’s medical needs and personal context” [6,7]. The unique relationship between patients and their family physicians may enhance continuity by fostering trust during difficult periods such as the end of life [6]. This complements our understanding of EOL outpatient visit concentration with family physicians.

Although continuity is extensively studied in both quantitative and qualitative health services research, the relationship between a patient’s geography is often overlooked as a predictor for outpatient care use, and subsequently continuity. Specifically, health services research has typically only considered patients rurality status, by means of categorical variables demarcating whether patients reside in urban or rural regions. For example, our previous study aimed at identifying the patient-level characteristics associated higher continuity during the last year of life, only included the patients’ residential rurality status (as a dichotomized variable measuring if a patient resided in an urban or rural region) as a predictor [8]. Other approaches that have been used are the Population Centre (POPCTR) method, the Statistical Area Classification (SAC), or the Rurality Index of Ontario (RIO) score [9]. The POPCTR method categorizes census metropolitan areas and census agglomerations into three categories (small, medium, and large urban centres), based on the population size and density of the census geographic unit. The SAC method also categorizes census boundaries into classifications; however, it is based on the percentage of the workforce that is composed of residents. Lastly, the RIO score is the most appropriate for healthcare as it produces a continuous score derived from population and population density, the travel time to the nearest basic referral centre (i.e., general inpatient hospitals), and the travel time to the nearest advanced referral centre (i.e., cancer care centres) [10]. While these approaches provide rich information regarding the effects of rurality on care use, they do not consider the impacts of patients’ geographic proximity to their family physicians as they do not measure the distance between the patients and their corresponding family physician at the individual-level.

### 1.2. Research gap

There is a significant evidence gap regarding the relationship patients’ geographic proximity to their family physicians and their EOL care. For example, Kelly et al. conducted a systematic review of 108 studies from high-income countries examining the association between travel time or distance to healthcare providers and health outcomes. None of these studies focused specifically on the EOL context, which is concerning as this period of life is often associated with an escalation of care [5]. Moreover, only three studies examined distance to family physicians, and all were conducted in the United States. Specifically, one study assessed the relationship between driving time to family physicians and timely cancer diagnosis and treatment [11], while the other two explored proximity to family physicians and health outcomes for patients with diabetes [12,13].

Although these studies did not address EOL care, Kelly et al.’s review identified a consistent ‘distance decay’ effect, where patients living further from their healthcare providers experienced poorer outcomes compared to patients who lived closer. Similarly, a separate literature review of 27 studies on travel burden for cancer diagnosis and treatment also found evidence of distance decay associations [14], yet only one study is from Canada [15]. Overall, very few studies have examined the proximity and healthcare use in the Canadian context, and most have focused on outcomes typically associated with specialist or inpatient care. To date, no studies have specifically investigated this relationship in the Canadian EOL setting.

### 1.3. Study objectives and hypothesis

The study objectives include: 1) to measure the association between the patients’ geographic proximity to their family physician and their relational COC with their family physician during the last year of life; and 2) to study the association between the patients’ geographic proximity to their family physician and relevant EOL healthcare outcomes.

This research will address a tangible aspect of access to care that is often simplified using rurality indicators and has not been explored in a Canadian context, despite the growing importance of family medicine access and the aging population. Understanding this relationship can help inform future health policy initiatives aimed at improving access to EOL healthcare. As such, we hypothesize that patients living closer to their family physician will experience higher continuity of care during the EOL and, as such, will also experience improved EOL healthcare outcomes.

## 2. Methods

### 2.1. Data sources

This study will used linked health administrative data from ICES, formerly known as the *Institute for Clinical Evaluative Sciences*. ICES is an independent, non-profit research institute funded by an annual grant from the Ontario Ministry of Health (MOH) and the Ministry of Long-Term Care (MLTC). As a prescribed entity under Ontario’s privacy legislation, ICES is authorized to collect and use health care data for the purposes of health system analysis, evaluation and decision support. Secure access to these data is governed by policies and procedures that are approved by the Information and Privacy Commissioner of Ontario.

### 2.2. Study design and population

This protocol was designed in alignment with the RECORD (Reporting of studies Conducted using Observational Routinely collected Data) statement [16]. We will use data from ICES to conduct a population-level retrospective cohort study of adult patients who died in Ontario, Canada, between January 1, 2021 and December 31, 2024. Cohort exclusion criteria include: 1) pediatric decedents (<19 years of age at death) to ensure that pediatric outpatient visits are not included in the analysis; 2) individuals with insufficient number of outpatient visits to their FP during the observation period (<3 visits) in order to calculate the relational COC indices; 3) individuals who changed residence during the exposure period, to mitigate geographic inconsistencies; 4) individuals ineligible for the Ontario Health Insurance Plan (OHIP) at any point in the exposure or observation periods; 5) admission to a long-term care facility within the last year of life as long-term care residents experience an institution-driven model of care where most of their care provision is provided by, often multiple, physicians attending their long-term care facilities; and 6) patients aged 105 years at death or older to prevent potential documented errors in date of birth information. As this will be a population-level analysis, all eligible individuals will be included in the cohort for analysis. Therefore, we can anticipate a very large sample size, as previous research identified approximately 360,000 decedents in Ontario between 2013 and 2017 [5].

### 2.3. Exposure definition

The primary exposure variable will be the patient’s geographic proximity to their formally rostered family physician at one year before death, measured in two units: metres (kilometres) and minutes (hours) (see section 2.6.1 Sensitivity Analyses for more detail).

First, the patient’s rostered family physician will be identified using the Client Agency Program Enrolment (CAPE) dataset, which records patients formally attached to a family physician under a capitation-based model. Patients without a formal attachment will be virtually rostered to the family physician with the greatest number of primary care-relevant OHIP billing codes in the two years preceding the last year of life [17].

Second, we will determine the patient’s most recent 6-digit postal code at one year before death using the Registered Persons Database and their corresponding family physician’s 6-digit postal code from the Corporate Physician Database. To minimize mis-classification due to large postal code boundaries, particularly in rural regions in Northern Ontario, we will use Statistics Canada’s Postal Code Conversion File+ (PCCF+) [18]. PCCF+ enables calculation of population-weighted centroids for each postal code boundary, by linking postal codes to all associated dissemination areas and applying population weights. This approach ensures that the identified centroids reflect actual residential distributions instead of the geometric centres of the boundaries – addressing the absence of individual-level address data for both patients and physicians due to privacy concerns. See below the equation for producing population-weighted centroids:


$$Weighted\;Latitude=\;\frac{\sum \left({Latitude}_{DA}\;\times \;{Population\;Weight}_{DA}\right)}{\sum {Population\;Weight}_{DA}}\;$$

$$Weighted\;Longitude=\;\frac{\sum \left({Longitude}_{DA}\;\times \;{Population\;Weight}_{DA}\right)}{\sum {Population\;Weight}_{DA}}$$

where *DA* are the individual dissemination areas linked to the postal code using PCCF+ and *Population Weight* is the proportion of the population within the dissemination area for that postal code.

Finally, we will use ArcGIS [19] Network Analyst with Ontario road, public transit, and walking path network data to calculate the shortest distance and travel time between the weighted centroid pairs [20]. The shortest routes across all three transportation modes will be defined as geographic proximity and will subsequently be categorized into empirical quartiles for analysis. This is a novel approach that provide a realistic measure of the travel burden experienced by patients instead of relying on the Euclidean distance (i.e., birds eyeview distance) between two geographic coordinates [21].

### 2.4. Outcome definitions

#### 2.4.1. Association between geographic proximity and EOL continuity.

We will use three relational COC indices (Appendix 1 in S1 File for their equations) when measuring the effect of geographic proximity to one’s family physician and continuity at the end of life:

1. **The Usual Provider of Care (UPC) index** measures the concentration of visits to a single provider out of all provider visits [22]. For this study, the rostered family physician will be pre-specified as the usual provider [8,23]. UPC scores range from 0 to 1, where 1 indicates all visits were to the rostered family physician.
2. **The Modified Bice-Boxerman (BB) index** expands on the unmodified BB index [24] by measuring the degree of the dispersion within each specialty instead of across specialties [25]. This index also ranges from 0 to 1, where 1 indicates all visits within each specialty were to the same physician.
3. **The relative variance index (RVI),** also known as the regularity index, measures the consistency of visit intervals to a single provider [26]. As such, we will measure the visit interval consistency with the rostered family physician and the index ranges from 0 to 1, where 1 indicates perfectly regular visit intervals.

All three indices will be calculated using outpatient visits during the patient’s last year of life, defined as all OHIP physician billing claims with location codes “Office”, “Phone”, or “Home”. Each index will also be categorized into empirical quartiles for analysis. All continuity index calculations will exclude billing claims from specialties not typically involved in the ongoing management of a patient’s condition, such as emergency medicine, anesthesiology, or pathology. Appendix 2 in S1 File provides a list of physician specialties to be excluded.

#### 2.4.2. Association between geographic proximity and EOL healthcare outcomes.

To study the impact of geographic proximity on EOL healthcare outcomes, the following outcomes will be studied:

1. **Number of days spent in the community** during the last year of life and the last 90 and 30 days of life.
2. **Referral to palliative home care services** during the last year of life and the last 90 and 30 days of life.
3. **Place of death,** categorized as acute care facility, sub-acute facility, or community.

Number of days spent in the community will be captured using Inpatient hospitalizations will be identified using the Discharge Abstract Database (DAD), ED visits using the National Ambulatory Care Reporting System (NACRS), and place of death using an ICES-derived macro [27,28] that considers multiple administrative datasets: 1) acute care deaths: DAD, NACRS, Same Day Surgery (SDS); 2) sub-acute deaths: Continuing Care Reporting System – Complex Continuing Care (CCRS-CCC), National Rehabilitation System (NRS), Ontario Mental Health Reporting System (OMHRS); and 3) community deaths: all other locations not captured in the previously holdings.

### 2.5. Covariates

Several patient-level demographic and clinical characteristics will be captured at one year before death (unless otherwise specified):

1. **Age** may affect the number of visits a patient may have in the last year, with evidence to suggest that seniors are more likely to visit their family physician [29]. Additionally, there is the possibility that younger patients move away further from the family physician due to increased costs of living, especially in large urban regions [30].
2. **Sex** will be included as there is evidence that female patients access more EOL care [31] and experience higher EOL COC [8], compared to males.
3. **Rurality status** will be included previous research also identified that patients living in urban regions were more likely to receive EOL care [31], yet patients living in rural regions were more likely to experience higher EOL COC [8].
4. **Socioeconomic status,** as patients with lower socioeconomic status may have decreased access to primary care physicians by relying on fee-for-service walk-in clinics over formal primary care teams.^28^
5. **Comorbidity status** will be measured using the number of prevalent Johns Hopkins Adjusted Clinical Groups [32], as patients with higher comorbidity burden may require more visits with their providers during at the end of life [31].
6. **Cancer** status will be measured as whether patients received any active cancer treatments (i.e., chemotherapy [33–36], surgeries [37,38], or radiation therapy [36]) during the last year of life. We want to control for active cancer treatment as patients dying of cancer receive greater access to palliative care at the end of life compared to patients who die of other conditions [8,23,31].

### 2.6. Statistical analysis

Patient demographic and clinical characteristics will be summarized using means and standard deviations for normally distributed continuous variables, or medians with interquartile ranges (25^th^ and 75^th^ percentiles) for non-normally distributed variables. Counts and percentages will be reported for categorical variables. Baseline characteristics will be stratified by quartiles of geographic proximity to assess differences across exposure levels. Differences will be tested using independent samples t-tests and Pearson’s Chi-square tests for continuous and categorical variables, respectively. Effect sizes will also be measured using Cohen’s *d* and Cramer’s *V* for continuous and categorical variables, respectively.

Study outcomes will be summarized using means (standard deviations), medians (interquartile ranges), and counts (percentages) across the entire cohort and stratified by exposure quartiles. Next, we will use regression analysis to examine associations between geographic proximity and study outcomes, adjusting for all previously specified covariates:

1. **Continuity outcomes (quartiles):** Cumulative link proportional-odds ordinal regression.
2. **EOL healthcare outcomes (count-based):** Poisson regression, or negative binomial regression with a log-link function if overdispersion is present.
3. **Place of death (categorical):** Multinomial logistic regression with a logit-link.

Statistical significance will be assessed at p < 0.001 (two-tailed) to account for the anticipated large sample size. All effect sizes and model coefficients will be reported with 95% confidence intervals, and all analyses will be conducted using Python [39].

#### 2.6.1. Sensitivity analyses.

We will repeat regression analyses using geographic proximity measured as travel time (i.e., minutes or hours) rather than distance (i.e., kilometres) to capture additional dimensions of travel burden, including potential traffic-related delays on popular travel routes.

### 2.7. Study status and timeline

This study is a retrospective cohort analysis using routinely collected health administrative data for the purpose of evaluating, monitoring and planning of the health care system. Therefore, participant recruitment is not applicable. Data collection involves the extraction and linkage of deidentified health administrative data; data collection and analysis has not yet commenced as of November 2025 Results from are expected to be available by May 2026.

## 3. Data management and quality assurance

Administrative datasets will be accessed using server connections and all linked datasets will be held in coded format in a secure research environment at ICES McMaster. Only the lead investigator and analytic epidemiologist at ICES McMaster will have access to the linked datasets to be used for analysis and will be responsible for data management and retention. However, the Python programming scripts for the analysis will be available upon request for potential replication analyses.

## 4. Ethics and privacy

Research projects that only use data collected by ICES are exempt from research ethics board review under section 45 of Ontario’s Personal Health Information Protection Act. Section 45 authorizes ICES to collect personal health information, without consent, for analyzing data to manage, evaluate, or monitor, the healthcare system. The use of this data is also approved by ICES’ Privacy and Legal Office. Therefore, this study does not require individual consent as this would also be pragmatically impossible given the study cohort of interest. In addition, the use of existing and routinely collected data poses minimal risk of harm to patients included in the study cohort. Further, ICES enforces a rigorous risk identification process when exporting research outputs to ensure that small cell issues are suppressed in research outputs, to eliminate the risk of re-identification.

## 5. Strengths and limitations

This study design has several strengths. First, this study utilizes a novel methodology, specifically geographic network analysis, which is not extensively used in health services research in Ontario. Geographic network analysis enables us to capture the individual-level travel burden patients experience, and in turn, providing a much more nuanced measure of travel burden or geographic access compared to the existing approaches commonly used in health services research. Second, the use of population-level data has several strengths, such as improving internal validity by capturing all documented healthcare interactions, ensuring that a wide variety of patients, such as both low and high healthcare users, are captured. Additionally, using population-level data increases the external validity of our potential results as we will include virtually all decedents from Ontario Between 2021 and 2024. Therefore, our potential findings may also be relevant to other jurisdictions with universal healthcare systems that provide robust family medicine services.

However, using population-level health administrative data will also have limitations as well. First, there will always be a certain degree of residual confounding by using health administrative data. Key personal information such as the patient’s preferred or available transportation methods cannot be determined. Similarly, we cannot assess if a patient experiences mobility issues that forces them to use a specific transportation method, which may increase or decrease their travel burden. Second, there is a risk of misclassification bias and potential measurement error due to not being able to access both patients’ and physicians’ full geographic information (i.e., their full address) beyond the 6-digit postal code due to privacy and confidentiality legislation. We aim to mitigate this by using weighted population centroids within each 6-digit postal code boundary to approximate geographic proximity; however, the risk remains at the individual-level. Lastly, our analysis focuses specifically on patients’ geographic proximity to their family physicians, however, many patients may receive primary care from allied health professionals such as nurse practitioners. Therefore, our results will not assess the impact of allied health as this data is unavailable in health administrative data [40–42].

## 6. Conclusion

This study will generate novel insights into how geographic proximity to family physicians during the end-of-life period influences continuity of care and healthcare outcomes at this critical stage. By integrating advanced geospatial methodologies with validated continuity indices and population-level health administrative data, our design addresses a key gap in Canadian health services research. Findings will be submitted to peer-reviewed journals and presented at health policy–focused conferences to inform future planning and policy development. Through this work, we aim to promote equitable access to family medicine during the final stages of life, particularly for medically complex patients.

## Supporting information

S1 File Appendix. (DOCX)
