# Supplementary material for: Measuring geographic proximity and continuity with family medicine at end-of-life: Protocol for a population-level retrospective cohort study using Canadian Health Administrative Data
Source: PLoS One. 2025 Dec 4;20(12):e0336790. doi: 10.1371/journal.pone.0336790 (PMC12677476; doi:10.1371/journal.pone.0336790)
Supplement: S1 File — (DOCX) [file pone.0336790.s001.docx]

# 8. APPENDICES

**Appendix 1: Equations for the relational continuity of care indices used**

| **Index** | **Equation** | **Notation** |
| --- | --- | --- |
| Usual Provider of Care Index | $UPC=\max\left( \frac{n_{i}}{n} \right)$ | where $n_{i}$= the number of outpatient visits to the usual provider (rostered family physician) in the last year of life, and $n$ = the total number of outpatient visits to all physicians in the last year of life. |
| Modified Bice-Boxerman Index | $BB=\frac{(\sum_{i=1}^{p} n_{i}^{2} )-N}{(\sum_{i=1}^{s} n_{j}^{2} )-N}$ | where *n_i_* is the number of outpatient visits to the *i*th provider, *n_j_* is the number of outpatient visits within the *j*th speciality, and the overall number of outpatient visits, physicians, and specialties are given by *N, p,* and *s*, respectively. Specialities with less than two outpatient visits are excluded during the observation period to ensure meaningful interpretation of within-speciality continuation. |
| Relative Variance Index | $r= 1 / ( 1+\frac{sd \left( Days \right)}{Mean \left( days \right)}*100)$ | where $Days$ is the number of days between outpatient encounters with the usual family physician in the last two years of life. |

## Appendix 2: List of ICES Physician Database physician specialties to be excluded from continuity calculations

1. FP/Emergency Medicine
2. Anesthesiology
3. Clinical pharmacology & toxicity
4. Critical care medicine
5. Critical care medicine - pediatric
6. Diagnostic radiology
7. Emergency medicine
8. Emergency medicine - pediatric
9. Medical genetics and genomics
10. Nuclear medicine
11. General pathology
12. Anatomical pathology
13. Forensic pathology
14. Hematological pathology
15. Medical biochemistry
16. Medical microbiology
17. Neuropatholoy
18. MISSING
19. OUT OF PROVINCE
20. OUT OF COUNTRY
